# No Interrelation of Motor Planning and Executive Functions across Young Ages

**DOI:** 10.3389/fpsyg.2016.01031

**Published:** 2016-07-12

**Authors:** Kathrin Wunsch, Roland Pfister, Anne Henning, Gisa Aschersleben, Matthias Weigelt

**Affiliations:** ^1^Institute of Sport and Sport Science, University of FreiburgFreiburg, Germany; ^2^Department of Psychology, University of WürzburgWürzburg, Germany; ^3^Developmental Psychology, SRH Fachhochschule for HealthcareGera, Germany; ^4^Developmental Psychology, Saarland UniversitySaarbrücken, Germany; ^5^Department Sport and Health, University of PaderbornPaderborn, Germany

**Keywords:** anticipatory planning, end-state comfort effect, developmental disorders, child development, motor development

## Abstract

The present study examined the developmental trajectories of motor planning and executive functioning in children. To this end, we tested 217 participants with three motor tasks, measuring anticipatory planning abilities (i.e., the bar-transport-task, the sword-rotation-task and the grasp-height-task), and three cognitive tasks, measuring executive functions (i.e., the Tower-of-Hanoi-task, the Mosaic-task, and the D2-attention-endurance-task). Children were aged between 3 and 10 years and were separated into age groups by 1-year bins, resulting in a total of eight groups of children and an additional group of adults. Results suggested (1) a positive developmental trajectory for each of the sub-tests, with better task performance as children get older; (2) that the performance in the separate tasks was not correlated across participants in the different age groups; and (3) that there was no relationship between performance in the motor tasks and in the cognitive tasks used in the present study when controlling for age. These results suggest that both, motor planning and executive functions are rather heterogeneous domains of cognitive functioning with fewer interdependencies than often suggested.

## Introduction

Anticipatory motor planning accounts for future body postures at the end of goal-directed movements. In their everyday lives, people need to plan many movements in advance. When grasping a cup that is standing upside-down in the cupboard, most people use an uncomfortable thumb-down posture to grasp the cup, then turn it around and end in a comfortable thumb-up posture, which makes it possible to pour coffee into the cup. This anticipatory planning performance is a signification of the so called end-state comfort (ESC) effect (Rosenbaum et al., 1990).

The ESC effect was first observed and examined by Rosenbaum et al. (1990), who took this observation into laboratory. They designed the bar-transport-task, in which a horizontally oriented bar with one black and one gray end laid horizontally on two supports. This bar had to be placed on either a red or a blue target disc, placed to the right and to the left of the supports, respectively. Participants could grasp the bar either with an overhand-grip or an underhand-grip, using their right hand. Interestingly, they chose the comfortable overhand-grip only to place the right end of the bar on the target (irrespective of bar and target color), whereas they used the initially uncomfortable underhand-grip, when the left end of the bar had to be placed on the target. The flexible selection of the initial grasp type allowed participants to end the object manipulation in a comfortable thumb-up posture (as opposed to an awkward thumb-down posture), even if this meant to tolerate an awkward posture at the beginning of the action.

**Figure 1:**
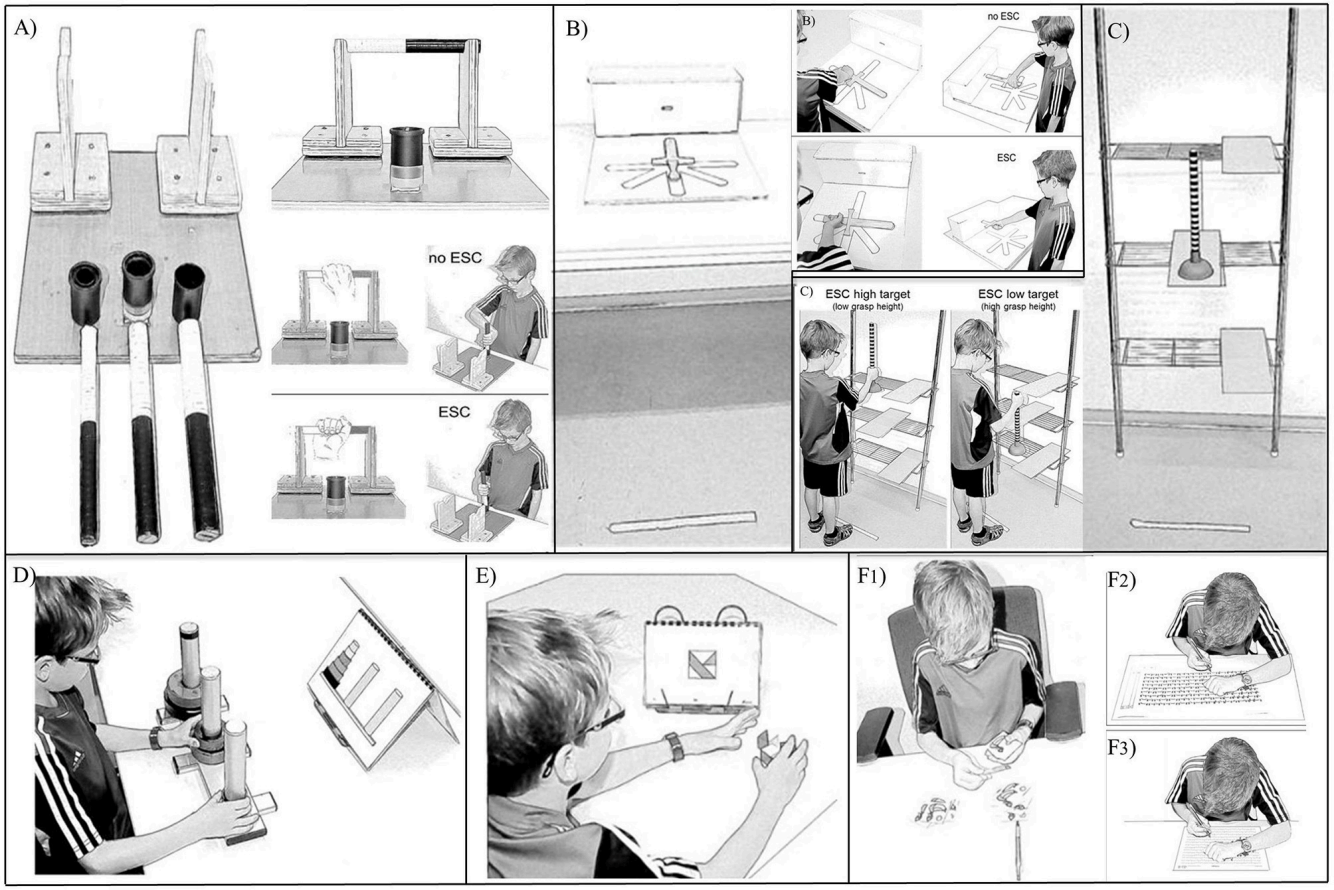
**Overview of the tasks used in the study and corresponding action outcomes for the motor tasks. (A)** bar-transport-task, **(B)** sword-rotation- task, **(C)** grasp-height-task, **(D)** Tower-of-Hanoi-task, **(E)** Mosaic-task, **(F**_1_**)** D2-task for preschoolers, **(F**_2_**)** D2-task for school children, **(F**_3_**)** D2-task for adults.

Since its discovery two decades ago (Rosenbaum et al., 1990), a growing body of research has documented the ESC effect as a robust phenomenon for healthy adults, as well as for different clinical populations (see Rosenbaum et al., 2012, for a review). Moreover, ESC effects also seem to arise in different non-human animals, (e.g., Zander et al., 2013). Similar anticipatory planning skills also become evident using other measures than the described bar-transport-task and its conceptual replications (see Figures 1A,B). One example for such additional measures is the grasp-height effect (Figure 1C). Here, anticipatory planning is probed by asking participants to put objects onto shelves of varying height. When placing objects on a high shelf, people grasp the object at its lower end. Conversely, when it has to be placed on a low shelf, they grasp the object at its upper end. Both actions result in a maximally comfortable position. Therefore, the future position of an object, which should be placed onto targets of varying heights, is also anticipated (Cohen and Rosenbaum, 2004).

For the following argument, we subsume all such measures under the umbrella term of ESC effects and, as noted above, these effects have been replicated numerous times (Rosenbaum et al., 2012). This, however, mainly concerns ESC effects for young adults, whereas only few studies investigated ESC effects in children (see Wunsch et al., 2013, for a review). Furthermore, the results of these latter studies were inconsistent with regard to onset of ESC effects during ontogenetic development: Whereas a number of studies suggested the ESC effect to occur already for 3-year olds in some task versions (Jovanovic and Schwarzer, 2011; Knudsen et al., 2012), increasing until the age of about 10 years (Thibaut and Toussaint, 2010; Jongbloed-Pereboom et al., 2013; Wunsch et al., 2015), other studies did not find evidence for anticipatory planning in 2- up to 7-year-old children (Smyth and Mason, 1997; Manoel and Moreira, 2005; Adalbjornsson et al., 2008; Van Swieten et al., 2010). The present study aimed at investigating a potential reason for these disparities in the literature by addressing the relation of ESC performance and executive functions, because differences in ESC performance might mirror differences in executive functioning, as outlined in the following.

The results of studies on motor planning and the ESC effect mentioned above indicate that humans, as well as non-human animals, grasp objects in a way that reflect their intentions. Therefore, studying grasping actions provides a window into internal planning processes. Motor planning, in turn, relies on cognitive control as can be inferred from clinical research. For example, it has been shown that motor deficiencies in people suffering from cerebral palsy do not only relate to movement execution, but also to motor planning (Mutsaarts et al., 2006). It can also be concluded from studies using dual-task paradigms. Here, simultaneously performing object manipulation and memory tasks showed a reduced recall ability, which suggests that planning for grasping objects needs (competes for limited) cognitive resources (e.g., Weigelt et al., 2009; Logan and Fischman, 2011; Spiegel et al., 2012). These findings seem to suggest a close link between anticipatory planning and executive functions (EF).

EF is an umbrella term that incorporates a collection of interrelated processes underlying purposeful, goal-directed behavior (Gioia et al., 2001). These executive processes are essential for the formation and maintenance of goals and strategies, preparation for action, and verification that plans and actions have been implemented appropriately (Luria, 1973). Results from studies using different EF tasks revealed that they can be explained in terms of three to four underlying factors (Levin et al., 1991; Welsh et al., 1991; Kelly, 2000). Based on these results, Anderson (2002) proposed a model of EF, which describes EF as four distinct domains: (1) attentional control, (2) information processing, (3) cognitive flexibility, and (4) goal setting. These functions are assumed to work in an integrative manner, in order to execute certain tasks, and that they can be conceptualized as an overall control system.

Executive processes further develop with the biological maturation of the frontal cortex throughout childhood and adolescence (Stuss, 1992). This development can be described as a multi-stage process, with different developmental trajectories for different functions (Passler et al., 1985). Therefore, EF play an important role in children's cognitive functioning, behavior, emotional control, and social interaction, but develop at different rates and at different times, differently for children's individual development. Accordingly, differences in grip selection between adults and children have been construed as indicating a deficit in children's planning skills (see Hughes, 1996; Smyth and Mason, 1997), with the presence of ESC as an indicator for “thinking ahead,” referring somewhat ambiguously to some kind of planning abilities. This led to an important debate, initially raised by Van Swieten et al. (2010), about whether performance in grip selection tasks is driven by executive planning (i.e., actively planning ahead to solve actions correctly or to avoid mistakes, for example) or motor planning (i.e., planning motor actions in advance in order to solve them correctly or most economically), or both (Stöckel et al., 2012; Scharoun and Bryden, 2013). Van Swieten et al. (2010) argued that the ESC effect cannot fully rely on executive planning, because adults do not consistently select grasps which end in comfortable positions. Thus, if executive planning was the driving force, executive functions would fail on some trials, but not on others, which is unlikely to be the case. Instead, Van Swieten et al. (2010) proposed that grip selection relies predominantly on pure motor planning processes and that the most efficient movement is selected for each grasp. On the other hand, it is commonly assumed that motor skills are very similar to intellectual skills in terms of acquisition and representation (Rosenbaum et al., 2001), which suggests the exact opposite of the aforementioned argument. In this regard, it has been assumed for a long time, that perceptual-motor skills and intellectual skills have closely related developmental origins, as already noted by Piaget (1952), who based the development of intelligence upon the emergence of skilled action.

A strong interrelation between perceptual-motor skills and intellectual skills also follows from embodied or grounded accounts of cognition and action (Barsalou, 2008; Borghi and Caruana, 2015). These accounts assume that behavioral decisions and cognitive operations alike are guided by modal simulations. Thus, even high-level functions, such as working memory, are assumed to be based on sensorimotor mechanisms (Borghi, 2005). In this perspective, executive functions would develop during, and because of, sensorimotor interactions with the environment. In contrast to the above-stated suggestion that EFs might resemble the driving force behind ESC-related motor planning, one could therefore argue that the development of executive functions might be driven by motor interactions with the environment instead. This prediction is not a necessary implication of embodied accounts, however: Once a particular function is developed, it may again influence more basic processes, such as motor planning. In any case, embodied accounts of cognition and action would propose a rather strong coupling of EFs and ESC performance.

Despite these theoretical arguments for a strong coupling of motor and cognitive skills, only few studies investigated this relationship directly. In a study conducted by Jenni et al. (2013), children between 7 and 18 years were tested, using the Zurich Neuromotor Assessment (ZNA) test and other, standardized intelligence tests, only weak correlations could be found between the performance in both, motor and cognitive tasks, which led the authors to suggest that motor and intellectual domains are largely independent. Another recent study conducted by Gonzalez et al. (2014) examined children between 5 and 10 years, using the Behavioral Rating Inventory of Executive Function (BRIEF) and two motor tasks with a focus on grasping. Results revealed significant correlations between the strength of right hand preference for grasping and numerous elements of the BRIEF, showing an interconnectedness of lateralization and EF. Moreover, Jansen (2014) conducted a review study, where she summarized results on studies on the relationship of motor activity and cognitive functions. She concluded that there is a positive effect of motor activity on the development of EF, and that specific physical activity can help to enhance specific cognitive functions in children. Altogether, motor (or better: physical) activity can play an important role in the development of EF and therefore, can act as a mediator on the relationship of ESC and EF.

Altogether, the question whether EF development may (at least partly) predict or influence the development of motor planning in terms of the ESC effect is still unanswered (see also Stöckel et al., 2012; Scharoun and Bryden, 2013). The similarity of the developmental trajectories of EF and the ESC effect suggests a potential relationship in their development (see Anderson, 2002, for the projected developmental trajectory of the different EF, and Wunsch et al., 2013, for an overview of the developmental trajectories for the different ESC tasks). Based on the assumption that ESC and EF are related, the present study investigated the possible role of executive functioning on the developmental trajectory of the ESC effect. To this end, we presented eight groups of children and a group of adults with three ESC tasks to assess their motor planning abilities and three EF tasks to measure their cognitive planning abilities. We predicted (1) an increase of task performance in each (ESC) subtest as children get older, (2) substantial inter-correlations between the ESC tasks, as well as, (3) positive correlations in each age group between participants' performance on ESC and EF tasks.

## Methods

### Participants

Nine age groups with a total of 217 participants were recruited. For a detailed overview of participant's demographics please see Table 1. All children were recruited from local daycare centers, elementary schools or via announcements in a local newspaper in Paderborn, Germany; all adults were students at the University of Paderborn. This study was carried out in accordance with the recommendations of the German Psychological Society (Deutsche Gesellschaft für Psychologie, DGPs). For all groups of children, parents provided their written informed consent for participation and for video recording their child during the experiment. All participants or their parents gave written informed consent in accordance with the Declaration of Helsinki. Participation was voluntary, without any financial compensation. Children received a personal certificate of participation and some sweets.

**Table 1:** **Demographic overview of the sample**.

**Age**	***M*_age_ (years) ± SD**	***N* (% of children)**	**Gender**		***M*_body−height_ (cm) ± SD**	***M*_table−height_ (% of body height)**	**Type of School**						**Handedness**	
			**♂**	**♀**			**Preschool**	**1st grade**	**2nd grade**	**3rd grade**	**4th grade**	**5th grade**	**Right**	**Left**
3-year-old	3.51 ± 0.32	21 (11.11)	10	11	101.76 ± 4.85	45.40	21	0	0	0	0	0	20	1
4-year-old	4.39 ± 0.24	23 (12.17)	11	12	109.78 ± 5.15	44.03	23	0	0	0	0	0	23	0
5-year-old	5.40 ± 0.26	26 (13.76)	13	13	115.77 ± 6.67	45.06	24	2	0	0	0	0	22	4
6-year-old	6.46 ± 0.29	22 (11.64)	15	7	123.91 ± 6.31	51.33	7	14	1	0	0	0	22	0
7-year-old	7.51 ± 0.33	26 (13.76)	11	15	130.69 ± 6.50	55.62	0	11	15	0	0	0	24	2
8-year-old	8.46 ± 0.29	27 (14.29)	11	16	134.52 ± 5.71	55.04	0	0	13	13	1	0	26	1
9-year-old	9.41 ± 0.30	23 (12.17)	10	13	141.09 ± 5.04	53.22	0	0	0	13	10	0	22	1
10-year-old	10.49 ± 0.31	21 (11.11)	8	13	147.19 ± 7.49	53.22	0	0	0	0	16	5	18	3
Adults	24.41 ± 2.21	28 (N/A)	15	13	176.29 ± 8.37	51.08	0	0	0	0	0	0	27	1

### Tasks and procedures

A test battery was designed to assess ESC planning and EF, consisting of three different tasks each: for the measurement of ESC we used (1) the bar-transport-task (Rosenbaum et al., 1990; Weigelt and Schack, 2010), in which participants were asked to grasp a horizontally oriented bar lying on two supports, with one black and one white end, and to insert one of its ends into a target hole in front of the support; (2) the sword-rotation-task (Rosenbaum et al., 1993; Crajé et al., 2010), in which participants had to insert a wooden sword, lying in different orientations in front of them on the table, into a target hole in a box behind it; and (3), for the first time in a child population, the grasp-height-task (Cohen and Rosenbaum, 2004; Weigelt et al., 2007), in which participants had to transport a vertically oriented dowel (a toilet plunger) from a chest-high platform either to a higher or to a lower platform. The EF tasks were chosen in accordance with the model of Anderson (2002): (1) the Tower-of-Hanoi-task (Simon, 1975; Welsh, 1991), which fits into each domain of the model, as feedback utilization, selective attention, planning abilities, strategic organization, and processing speed are needed. Here, participants had to build a tower of discs, as shown by a target position, following several rules with increasing difficulty as trial number increased; (2) the Mosaic-task (Wechsler, 1997, 2002, 2003), which fits into three of the domains, requiring feedback utilization, selective attention, and processing speed. In this task, a given mosaic pattern had to be re-created with a set of building blocks, and (3) the D2-attention-endurance-test (Brickenkamp, 1962), where selective attention, inhibition, and the speed of processing are necessary to achieve good results. Here, “d's” (or ducks in the children's version; Grob et al., 2009, 2013) with a given attribute had to be sketched (or sorted) out.

For each task, the respective materials were placed on a table in front of the participant, except for the grasp-height-task, for which the shelf was placed next to the table. Participants stood in front of the table for the bar-transport-task and for the sword-rotation-task, and in front of the shelf for the grasp-height-task. All EF tasks were performed at the same table, but this time with the participants sitting, while seat height was adjusted for each participant. Table height was 55 cm for kindergarten children and 75 cm for school children and adults. To adjust for differences in body height, children smaller than 110 cm stood on a 10 cm high podium, and children smaller than 120 cm or 130 cm stood on a 20 or 10 cm high podium in order to level the requirements in the ESC tasks. To control for differences in the relative table height compared to body height of participants, mean table heights were computed, indicating the relative table height compared to body height (including the podium height in percent) of participants (please see Table 1). Relative table height was between 44 and 55% of individual body height, meaning that the apparatus for the ESC tasks was located at body-center (±5%). A camera was positioned 150 cm besides the participant, at a height of 160 cm, and recorded the whole experiment for later coding.

Participants were tested individually by one experimenter. For several preschoolers, a teacher or a parent was also present in order to make the child feel comfortable. Prior to testing, adult participants or children's parents completed a short questionnaire on handedness, on how they completed their way to kindergarten/school/university, and on leisure time activities, sport participation, and spoken languages. Afterwards, children completed a short test to determine handedness as the hand that was used in at least two out of the three tasks (to throw a ball, use a spoon, and write/draw with a pencil). This hand was marked with an ink stamp. In the ESC tasks, children were instructed to always use the “stamp-hand.” The ESC tasks were run without familiarization trials. The order of all six tasks was randomized across participants. In the ESC tasks, participants stood in front of the table or the shelf, 10 cm away from the edges, respectively, or sat in front of the table with the respective apparatus on it for the EF tasks, with materials 10 cm away from the edge of the table. On average, the entire session lasted between 90 and 100 min. The duration differed according to participant's time needed to complete the different tasks and according to requests for breaks between the subtests. In general, most of the adults were able to complete the testing session in about 75 min, whereas some children needed up to 150 min to complete the whole session.

#### Motor tasks: assessing ESC

##### Bar-transport-task

A modified version of the original bar-transport-task (Rosenbaum et al., 1990) was used, which was similar to the one employed by Weigelt and Schack (2010). Different to the original task version by Rosenbaum et al. (1990), there was only one target at midline in front of the apparatus (and not two targets on either side as in the original task). A wooden bar, 20 cm long, with one black and one white end rested horizontally on two cradles, 15 cm above the table. A 5 cm high, black cylindrical container served as the movement target and was placed 10 cm in front of the support. To keep precision requirements comparable across age groups, the bar's diameter measured 1.5, 2, or 2.5 cm for preschoolers, school-children and adults, respectively, and the target hole's diameter measured 2, 2.5, or 3 cm for preschoolers, school-children and adults, respectively (see Figure 1A).

The start orientation of the bar (i.e., black or white end on the right side) was counterbalanced across participants and remained constant throughout the experiment. Participants were instructed to adopt the starting position (i.e., to stand behind the line with their hands facing their legs), to then grasp the bar firmly with their “stamp-hand,” and to insert the black or white end of the bar into the target hole, as indicated by the experimenter. After the insertion, they were instructed to return to the starting position. To prevent observational learning, the experimenter used a pincer grip at one end of the bar to reposition the bar back on the two cradles. Participants completed six trials in randomized order, three trials for each end. They could use either an overhand or an underhand grip to grasp the bar. This resulted in either an upright (thumb-up) or an inverted (thumb-down) hand position at the end of the movement. In the three uncritical trials, an overhand grip automatically resulted in a comfortable thumb-up position; in the three critical trials, however, an underhand grip was necessary to end in the comfortable thumb-up position and therefore, in ESC. Grip choice was coded from the video. Following recent studies (Adalbjornsson et al., 2008; Weigelt and Schack, 2010; Stöckel et al., 2012), we considered the ESC effect to be present if in a given condition (critical and uncritical trials) at least two out of the three trials ended in the comfortable position, resulting in a dichotomous outcome.

##### Sword-rotation-task

A variation of the original handle-rotation-task by Rosenbaum et al. (1993) was used, which was similar to the task versions created by Crajé et al. (2010) and by Jongbloed-Pereboom et al. (2013). A wooden sword (30 cm in length, 3.2 cm in width, and 0.8 cm in height; handle length = 10 cm) was horizontally placed on a platform (47 × 47 cm) in front of a target box in one of six start positions (Position 1 = 0⋅ (12 o'clock position), Position 2 = 90⋅, Position 3 = 135⋅, Position 4 = 180⋅, Position 5 = 225⋅, and Position 6 = 270⋅). The sword's blade had to be inserted into a tight fitting hole in a wooden block (47 cm in length, 16 cm in width, and 16 cm in height; hole: 3.5 × 1 cm; see Figure 1B). The same apparatus was used for all age groups.

Again, participants were instructed to adopt the starting-position and were told that they were a pirate (to adults this task was explained without the cover story) and that they had to insert the sword into the box by firmly grasping the handle in exactly the position it laid on the table, that is, without turning the sword before grasping it. Each session started with Position 1 to make sure participants understood the task. The experimenter retrieved the sword from the box and repositioned it on the platform always by grasping it at the cross guard to avoid observational learning. The task consisted of three blocks of 6 trials (one for each position), resulting in a total of 18 trials. Trial positions were randomized in each block. Participants could choose grips that resulted in either a comfortable end position (with the thumb pointing toward the blade) or an uncomfortable end position (with the thumb pointing away from the blade). Within each block, two trials were critical. Here, grasping the sword in a more uncomfortable hand position (at Positions 2 and 3) resulted in a comfortable end position. Grip choice was coded from the video. We considered the ESC effect to be present if at least 4 out of the 6 critical trials ended in a comfortable position platform (dichotomous outcome).

##### Grasp-height-task

This study is the first to examine the grasp-height effect in children. An adaptation of the original grasp-height-task by Cohen and Rosenbaum (2004) was used, which was similar to the version employed by Weigelt et al. (2007). Participants had to transport a standard toilet plunger from a home platform to a lower or higher target platform, and back to the home platform. The stem of the plunger was painted black and white in an alternating order, with a distance of 1 cm between each stripe. Board heights were individually adjusted to participants' body height by taking the board heights and average height of adult participants reported in Rosenbaum et al. (2006) as reference. For example, for an 87 cm tall child, shelf heights were 25.4, 43.2, and 61.0 cm for the low, middle and high shelf, respectively. On each shelf board, a wooden platform was attached in such a way that it protruded 15 cm from the shelf. The home platform was attached to the horizontal center of the middle shelf board. The two target platforms were attached to the participant's side of handedness, one on the low and the other one on the high shelf board. The toilet plunger stood on the home platform: a circular rubber base (10 cm in diameter and 5 cm high) supported the cylindrical wooden shaft (2.5 cm in diameter and 33 cm or 44 cm in length for child or adult participants, respectively, see Figure 1C).

Participants were told to adopt the starting position (standing behind the line, with hands facing their legs). Child participants were presented a cover-story suggesting that their performance was videotaped in this task in order to program a robot afterwards, which could then perform the same actions as they did. Participants' task was to stand in the start position, to grasp the plunger firmly on the shaft and to transport it to the platform indicated by the experimenter (home-to-target moves). Afterwards, they had to resume the starting position and were then instructed to bring the plunger back to the home platform (target-to-home moves). Participants were told that they needed to closely follow the instructions and move the plunger on the direct way to the named platform, and that home and target platform could differ across trials. A total of six trials had to be completed, with three movements to each of the target platforms. Conditions were blocked, with the start platform for the first three trials counterbalanced across participants. Grasp height was coded from the video by counting the distance from the plunger base to the hand of the participants. Participants showed planning for ESC if they grasped the plunger lower for the home-to-target moves than for the target-to-home moves for the high platform, and higher for the home-to-target moves than for the target-to-home moves for the low platform. ESC was considered to be present if participants showed this pattern in at least two out of the three trials for each platform (dichotomous outcome). Also, mean differences in grasp heights between home-to-target and target-to-home moves in the two conditions were computed and used for all correlation analyses. Ideal ESC performance would result in mean differences of zero, whereas positive or negative numbers indicate insufficient planning. For example, if the plunger was (initially) grasped slightly too high in the home-to-target moves for the upper target, it could then only be placed at the final position in a somewhat awkward body posture (greater stretch). Likewise, if the plunger was (initially) grasped slightly too low in the home-to-target moves for the lower target, it could only be placed while bending the upper body, which is also more uncomfortable.

#### Cognitive tasks: assessing EF

##### Tower-of-Hanoi-task

We used a slightly modified version of the original task (Simon, 1975), which was similar to Welsh (1991), but used only one apparatus as in the original version. It consisted of 3 pegs in a row (height: 23 cm, diameter: 4 cm, distance between pegs: 15 cm) that were attached on a bottom plate. On these pegs, up to five discs of varying size and color could be located: black (diameter: 13 cm), blue (diameter: 11 cm), green (diameter: 9 cm), red (diameter: 7 cm) and yellow (diameter: 5 cm). The target position was indicated by a picture, displayed at a 75⋅ angle on a music stand, 20 cm behind the apparatus (see Figure 1D). The peg on the very right was the target peg, and was marked with a black duct tape at the top.

Participants were instructed to build a tower of discs as shown by the target position on the picture, starting from the arrangement presented. Each participant was to solve up to 10 different tower problems, with increasing degree of difficulty. Depending on the number of discs and on the starting arrangement, trials differed in the minimal number of moves necessary to complete the tower. Three test versions were created with difficulty adjusted to age: a version for 3- and 4-year-olds, for 5- and 6-year-olds, and for 7-year-olds and older children and adults. Every test started with a familiarization trial with three discs (2, 3, or 6 moves according to the test version), in which rules were explained and questions could be clarified. Participants had to follow three rules: (1) move only one disc at a time, (2) a disc may only be in your hand or on a peg, but not on the table or somewhere else, and (3) a smaller disc can be placed on top of a bigger disc, but a bigger disc cannot be placed on top of a smaller disc. In test trials, each starting position was initially covered by placing a cardboard in front of the apparatus to assess latency. The task was terminated whenever participants were not able to solve a tower problem in up to twice the minimal number of moves necessary to solve the problem, or if participants did not move any disc for more than 90 s. The single tower problems included 3 or 4 discs in the two easier test versions, and 4 or 5 discs in the most difficult version. Minimal number of moves necessary ranged between 2 and 15 for 3- and 4-year-olds, between 4 and 15 for 5- and 6-year-olds, and between 7 and 31 for children aged 7 and older, and adults^1^. Start- and end-positions of the discs were checked from the video and the number of steps to complete the tower was counted. Number of tower problems completed correctly served as dependent measure.

##### Mosaic-task

This task is a subtest of the Wechsler Scale of Intelligence^2^. In the Mosaic-task, a given mosaic pattern has to be re-created with a set of building blocks. The target picture or the 3D model was positioned 18 cm away from the edge of the table, 10 cm to the side of participants' body midline on the opposite side of handedness. Participants had to arrange up to nine cubes (side length: 2.5 cm) of different colors (all red sides, all white sides or red and white sides) (see Figure 1E). The test was administered in accordance with the test manual and age group. Completion time was coded from the video and performance was checked for accuracy. The percentage of scored points was calculated as indicated in the test manual.

##### D2-attention-endurance-test

Three versions of this speeded test of selective attention (Brickenkamp, 1962) were used: the analogous subtests in the Intelligence and Development Scales for preschool children (IDS-P; Grob et al., 2013) and for school children (IDS, Grob et al., 2009), and the D2-R for adults (Brickenkamp et al., 2010). Preschool children's task was to sort cardboard cards (6 × 6 cm) showing a duck, according to the presence or absence of a distinct characteristic. A pencil, lying 25 cm away from the child at the side of their handedness indicated where to stack the cards with the given characteristic (see Figure 1F_1_). In the paper-pencil version for school children, participants were presented with a DIN A3 sheet of paper showing rows of ducks with or without distinct characteristics. Children had to mark the ducks with the target characteristics (see Figure 1F_2_). In the paper-pencil version for adults, rows of the letters *p* and *d* were presented on a DIN A4 sheet of paper, and letters with distinct characteristics had to be marked (see Figure 1F_3_). Videos of preschool children were checked to verify the number of properly sorted cards. Performance was scored in accordance with the respective test manual. According to the test manual, the total number of scored points served as dependent measure.

### Data analysis/scoring

Chi-Square tests were used to examine group differences in the ESC tasks due to the dichotomous nature of the dependent variables. For the EF tasks^3^, comparison across age groups is difficult due to the usage of different task versions and therefore are not reported in the results section. To test for age effects on ESC performance, we further conducted regression analyses on the mean percentage of participants who showed ESC in each age group. Finally, bivariate Pearson's correlations were computed in order to find possible relationships within the ESC tasks and the EF tasks, and between these motor and cognitive tasks. It should be noted that, for the current sample sizes, the individual correlations within each age group come with sufficient power only for large correlations, whereas the assessment across all age groups ensures sufficient power also to detect rather small effect sizes (1-β = 0.80 for *r* = 0.19 with two-tailed tests, as determined via the pwr package in R; Champely, 2015). The findings within each group should thus be treated with caution, whereas there is good reason to interpret the overall finding of a null-correlation as evidence for a true null effect (rather than a Type II error).

## Results

### The development of ESC

#### Bar-transport-task

**Figure 2:**
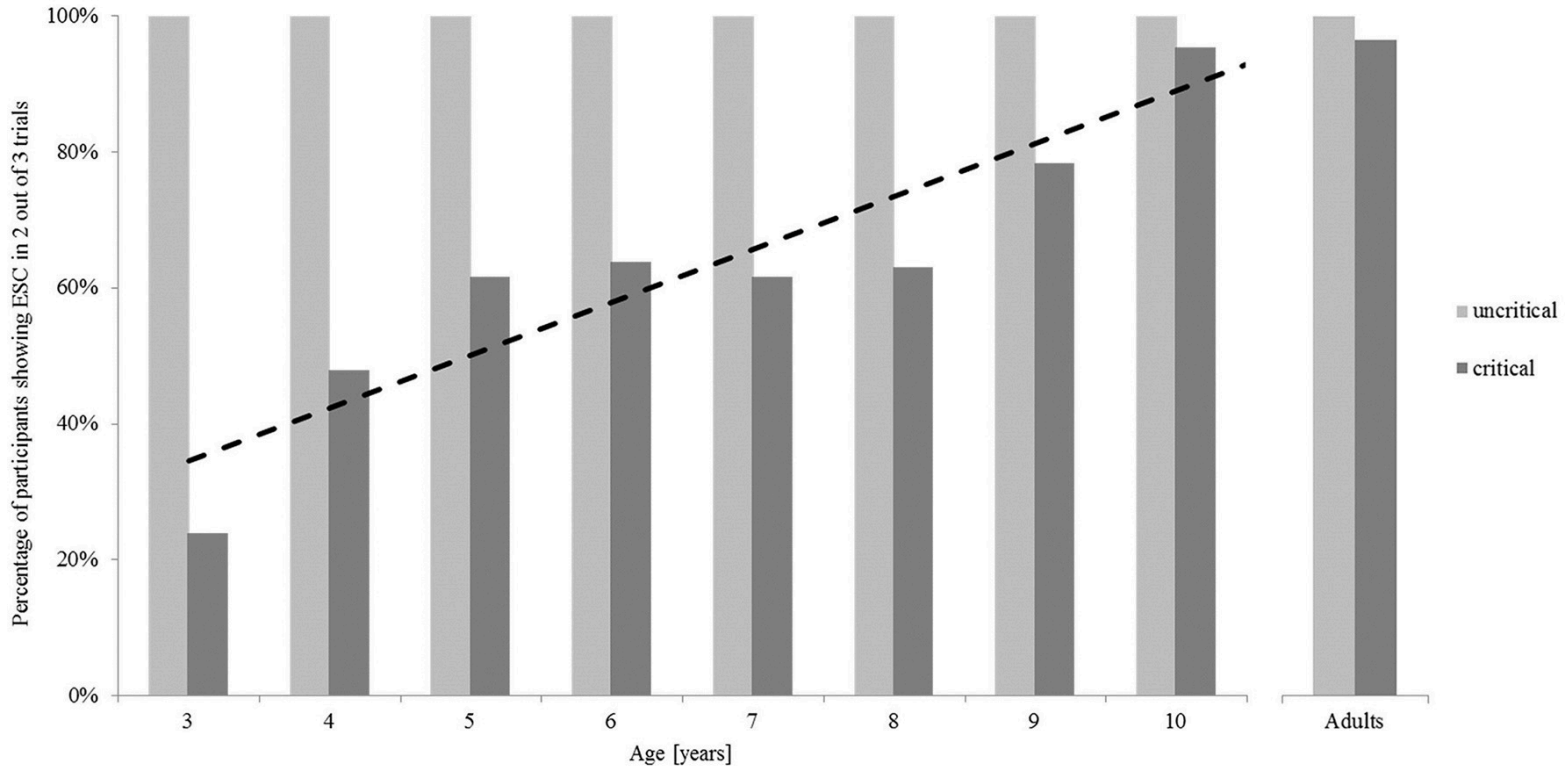
**Percentage of the participants in each group showing end-state comfort in at least two out of three trials in the uncritical (initial overhand grip) and critical (initial underhand grip) conditions in the bar-transport-task**.

Figure 2 illustrates the mean percentage of participants in each group who showed ESC planning. In the uncritical trials, all participants in all age groups adopted an overhand grasp in at least two out of the three uncritical trials and therefore ended in a comfortable end position. In the critical trials, only 24% of the 3-year old children showed sensitivity for ESC planning. This amount increased up to 62% in the 5-year-olds (see Table 2). Here, a stagnation of the developmental trajectory can be seen in 5-to-8-year old children, with a mean of 63% showing ESC planning. Then, the percentage of participants showing ESC increased again up to 95% in the 10-year-olds, which is comparable to adult behavior. A chi-square analysis showed these differences in the proportion of children showing ESC in the critical trials to be significant, $\chi_{\left(7\right)}^{2}$ = 34.93, *p* < 0.001.

**Table 2:** **Detailed results of all age groups in the six different tasks**.

	**Bar-transport-task**		**Sword-rotation-task**		**Grasp-height-task**			**Tower-of-Hanoi-task**	**Mosaic-task**	**D2-task**
	**(% of participants)**		**(% of participants)**		**(% of participants)**		***M*_differencce_ in grasp height (cm)**	**(% of solved items)**	**(% of points)**	**(% of points)**
	**Uncritical**	**Critical**	**Uncritical**	**Critical**	**HtT-moves**	**TtH-moves**				
3-year-olds	100.00	23.81	100.00	42.86	19.05	4.76	−15.15	12.86	37.58	36.31
4-year-olds	100.00	47.83	100.00	43.48	34.78	4.35	−14.71	23.91	48.47	47.52
5-year-olds	100.00	61.54	100.00	57.69	19.23	7.69	−12.92	45.77	71.15	28.12
6-year-olds	100.00	63.64	100.00	63.64	31.82	0.00	−10.77	53.64	55.05	36.56
7-year-olds	100.00	61.54	100.00	73.08	38.46	3.85	−8.61	41.92	51.38	45.38
8-year-olds	100.00	62.96	100.00	74.07	33.33	22.22	−5.35	47.78	69.00	52.54
9-year-olds	100.00	78.26	100.00	78.26	39.13	26.09	−4.66	49.13	75.24	64.37
10-year-olds	100.00	95.24	100.00	76.19	45.45	13.64	−3.51	56.67	85.51	71.35
Adults	100.00	96.43	100.00	100.00	92.86	50.00	4.68	66.07	67.70	43.12

*To be considered as showing ESC, participants needed to meet the following requirements: show ESC in at least 2 out of 3 trials in the bar-transport-task, in at least 4 out of 6 trials in the sword-rotation-task, and in at least 2 out of 3 moves to either platform in the grasp-height-task. HtT, Home-to-Target-moves; TtH, Target-to-Home-moves*.

A regression analysis of the percentage of participants showing ESC across the child groups revealed the developmental trajectory in the critical trials to be statistically significant with an increase of 7.8% per year, *t*_(6)_ = 5.622, *p* = 0.001. The entire regression model including the intercept yielded an adjusted *R*^2^ = 0.814, *F*_(1, 7)_ = 31.608, *p* < 0.001. Single chi-square four-field tests revealed that all children groups up to the age of 9 years behaved significantly less often in terms of ESC than adults did; $\chi_{\left(1\right)}^{2}$ = 27.93, *p* < 0.001 for the 3-year-olds; $\chi_{\left(1\right)}^{2}$ = 15.71, *p* < 0.001 for the 4-year-olds; $\chi_{\left(1\right)}^{2}$ = 10.12, *p* < 0.01 for the 5-year-olds; $\chi_{\left(1\right)}^{2}$ = 8.98, *p* < 0.01 for the 6-year-olds; $\chi_{\left(1\right)}^{2}$ = 10.12, *p* < 0.01 for the 7-year-olds; $\chi_{\left(1\right)}^{2}$ = 9.62, *p* < 0.01 for the 8-year-olds; $\chi_{\left(1\right)}^{2}$ = 4.02, *p* < 0.05 for the 9-year-olds). There was no difference in behavior between 10-year-olds and adults [$\chi_{\left(1\right)}^{2}$ = 0.04, *p* > 0.05].

**Figure 3:**
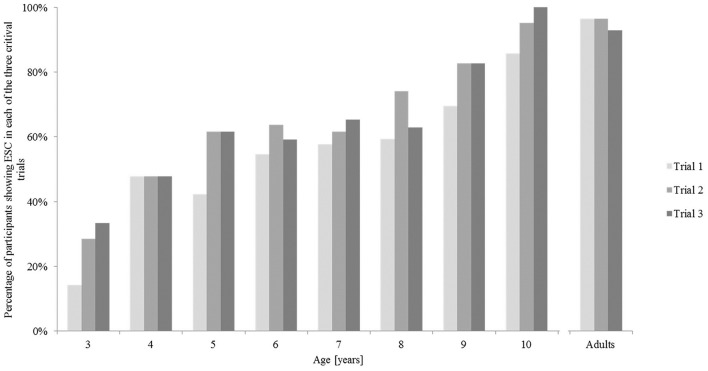
**Percentage of participants showing end-state comfort in each of the three critical trials in the bar-transport-task**.

Considering all children, a Kruskal-Wallis Test revealed the differences between the age groups to be statistically significant across all three trials [Trial 1 $\chi_{\left(7\right)}^{2}$ = 28.62, *p* < 0.01; Trial 2 $\chi_{\left(7\right)}^{2}$ = 27.71, *p* < 0.001; Trial 3 $\chi_{\left(7\right)}^{2}$ = 26.76, *p* < 0.001]. This shows the distinct developmental trajectory for ESC planning. Trial repetition data (i.e., whether children change their grip behavior across the trial repetitions and thus, exhibit short-term learning effects over the course of the three critical trials), is depicted in Figure 3.

Concerning these results, there is no systematic pattern detectable, suggesting that short-term learning effects may rather not have occurred across the three trials.

#### Sword-rotation-task

**Figure 4:**
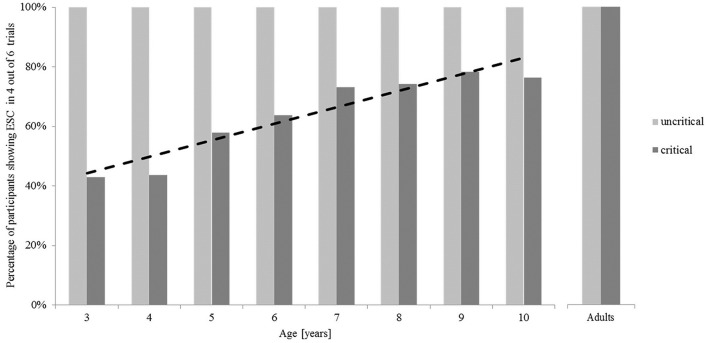
**Percentage of participants in each group showing end-state comfort in the sword-rotation task in at least seven out of the 12 uncritical trials and in at least four out of the six critical trials**.

Figure 4 illustrates the mean percentage of participants in each group who showed ESC planning for both conditions. In the uncritical trials, all participants adopted a grip with the thumb being oriented toward the blade, and thus, ended in a comfortable position when inserting the sword into the box. In the critical trials, however, only 43% of the 3- and 4-year old children showed sensitivity for ESC planning. This amount increased up to 64% in the 6-year-olds. Again, similar to the bar-transport-task, even if more delayed, a stagnation of the developmental trajectory can be detected in 7-to-10-year old children, with a mean of 75% of the children showing ESC. All adults showed ESC in the six critical trials (see Table 2). A chi-square analysis showed these effects in the proportion of children showing ESC in the critical trials to be marginally significant, $\chi_{\left(7\right)}^{2}$ = 12.89, *p* = 0.075.

A regression analysis of the percentage of participants showing ESC revealed the developmental trajectory in the critical trials to be statistically significant with an increase of ESC conform grasps of 5.5% per year, *t*_(6)_ = 7.156, *p* < 0.001, adjusted *R*^2^ = 0.878, *F*_(1, 7)_ = 51.209, *p* < 0.001. Single chi-square four-field tests revealed that all children groups behaved significantly less often in terms of ESC than adults did; $\chi_{\left(1\right)}^{2}$ = 21.19, *p* < 0.001 for the 3-year-olds; $\chi_{\left(1\right)}^{2}$ = 21.24, *p* < 0.001 for the 4-year-olds; $\chi_{\left(1\right)}^{2}$ = 14.88, *p* < 0.001 for the 5-year-olds; $\chi_{\left(1\right)}^{2}$ = 12.12, *p* < 0.001 for the 6-year-olds; $\chi_{\left(1\right)}^{2}$ = 8.66, *p* < 0.01 for the 7-year-olds; $\chi_{\left(1\right)}^{2}$ = 4.24, *p* < 0.05 for the 8-year-olds; $\chi_{\left(1\right)}^{2}$ = 6.75, *p* < 0.01 for the 9-year-olds; and $\chi_{\left(1\right)}^{2}$ = 7.42, *p* < 0.01 for the 10-year-olds).

**Figure 5:**
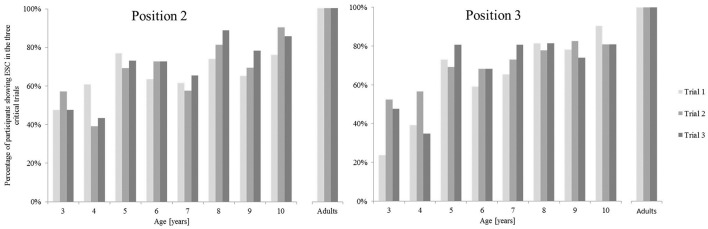
**Percentage of the participants showing end-state comfort in each of the three critical trials for Positions 2 and 3, respectively**.

Considering all children, a Kruskal-Wallis Test did not consistently reveal the differences between the age groups to be statistically significant across all three trials in Position 2 [Trial 1 $\chi_{\left(7\right)}^{2}$ = 7.22, *p* > 0.05; Trial 2 $\chi_{\left(7\right)}^{2}$ = 10.47, *p* > 0.05; Trial 3 $\chi_{\left(7\right)}^{2}$ = 15.58, *p* < 0.05] and in Position 3 [Trial 1 $\chi_{\left(7\right)}^{2}$ = 29.97, *p* < 0.001; Trial 2 $\chi_{\left(7\right)}^{2}$ = 11.44, *p* > 0.05; Trial 3 $\chi_{\left(7\right)}^{2}$ = 16.95, *p* < 0.05]. We also investigated whether children change their grip behavior across the trial repetitions and thus, exhibit short-term learning effects over the course of the six critical trials. Figure 5 depicts the percentage of children in each age group performing in a manner consistent with second-order motor planning in each of the three critical trials, in the left for Position 2, in the right for Position 3.

A closer examination of trial repetition data did not yield a systematic pattern. It is not the case that children started to plan for ESC with increasing trial number. Therefore, like in the bar-transport-task, short-term learning effects are unlikely to have improved participants' performance in terms of ESC.

#### Grasp-height-task

Results showed, that the percentage of participants showing ESC in the high target moves increased from 19% in the 3-year-olds up to 45% in the 10-year-olds, whereas 93% of the adults showed ESC planning. In the low target moves, the percentage of ESC conform grasps increased from 5% in the 3-year-olds up to 26% in the 9-year-olds, with 10-year-olds showing less ESC. Interestingly, this developmental pattern does not follow a linear increase, with older children sometimes performing poorer than younger ones (see Table 2). Fifty percent of all adults showed ESC like planning. Thus, ESC planning seems to be more frequent for bringing the object to high positions than to low positions. But, even 10-year-old children showed only half as many grasp behaviors in terms of ESC as adults do. This hints to a rather late emergence of ESC planning for tasks exploiting a continuous task space.

A chi-square analysis revealed significant differences between the groups in the amount of participants showing ESC averaged across both positions [$\chi_{\left(7\right)}^{2}$ = 120.59, *p* < 0.001]. A regression analysis of the mean differences in grasp height across the child groups revealed the developmental trajectory to be statistically significant with a slope of 1.81 cm per year, *t*_(6)_ = 12.986, *p* < 0.001, adjusted *R*^2^ = 0.878, *F*_(1, 7)_ = 166.011, *p* < 0.001. Single chi-square four-field tests revealed that all children groups behaved significantly less often in terms of ESC than adults did [$\chi_{\left(1\right)}^{2}$ = 34.24 for the 3-year-olds; $\chi_{\left(1\right)}^{2}$ = 27.21 for the 4-year-olds; $\chi_{\left(1\right)}^{2}$ = 36.86 for the 5-year-olds; $\chi_{\left(1\right)}^{2}$ = 30.49 for the 6-year-olds; $\chi_{\left(1\right)}^{2}$ = 27.34 for the 7-year-olds; $\chi_{\left(1\right)}^{2}$ = 20.95 for the 8-year-olds; $\chi_{\left(1\right)}^{2}$ = 15.32 for the 9-year-olds; and $\chi_{\left(1\right)}^{2}$ = 17.70 for the 10-year-olds, all *p* < 0.001].

**Figure 6:**
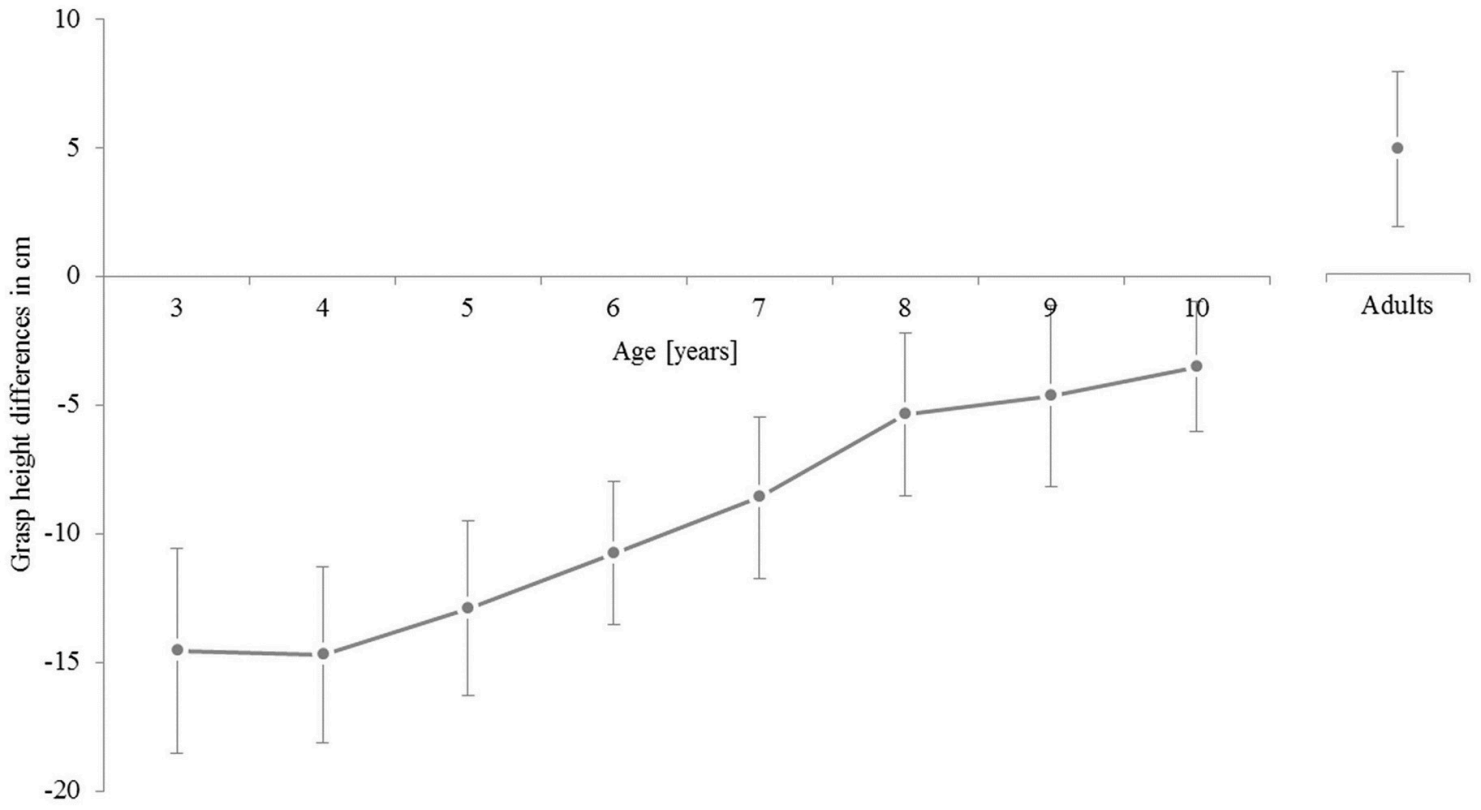
**Mean differences in grasp height averaged for the high-target-moves and the low-target-moves**. Error bars represent 95% confidence intervals for the individual means.

For further analysis, a score was computed for the mean differences in grasp height across both target positions (see Figure 6). First, the mean grasp heights for home-to-target and for target-to-home moves were computed for both, high target and low target trials. For both, the differences of the mean grasp heights were computed. The means from these two differences were multiplied by (−1). This resulted in the distribution shown in Figure 6.

**Figure 7:**
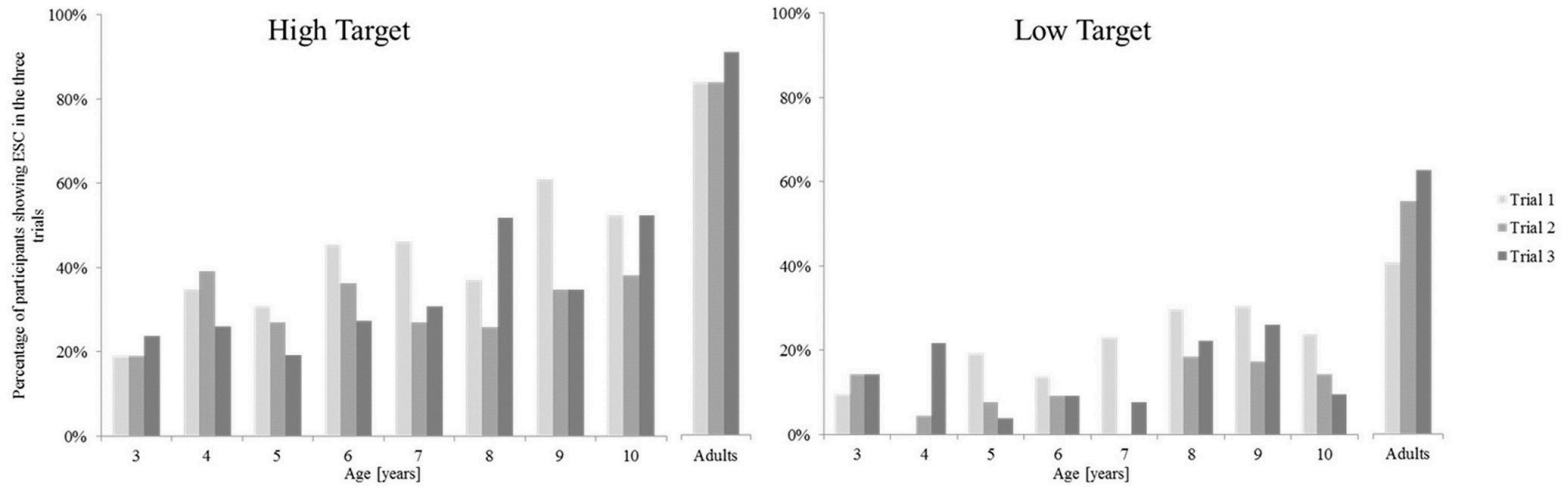
**Percentage of participants showing end-state comfort in each of the three trials for the high and the low target platform**.

Considering all children, a Kruskal-Wallis Test revealed the differences between the age groups to be statistically significant in Trial 1 and Trial 3 for the high target platform [Trial 1 $\chi_{\left(7\right)}^{2}$ = 17.07, *p* < 0.05; Trial 2 $\chi_{\left(7\right)}^{2}$ = 11.37, *p* > 0.05; Trial 3 $\chi_{\left(7\right)}^{2}$ = 22.09, *p* < 0.01] and in all trials for the low target platform [Trial 1 $\chi_{\left(7\right)}^{2}$ = 21.78, *p* < 0.01; Trial 2 $\chi_{\left(7\right)}^{2}$ = 25.48, *p* < 0.001; Trial 3 $\chi_{\left(7\right)}^{2}$ = 28.98, *p* < 0.001]. Again, it was investigated whether participants change their grasp height across the trial repetitions and thus, exhibit short-term learning effects over the course of the six trials. Figure 7 depicts the percentage of children in each age group performing in a manner consistent with second-order motor planning in each of the three trials, in the left graph for the high target platform, in the right graph for the low target platform.

The results show a similar pattern as observed in the other ESC tasks, as there was no systematic pattern of ESC development in grasp height. Therefore, like in the bar-transport-task and in the sword-rotation task, short-term learning effects are unlikely to have improved participants' performance in terms of ESC.

In summary, Table 2 provides a detailed overview over the results of all age groups in the six different tasks.

### Intercorrelations of the ESC tasks

The results above suggested similar developmental trends for ESC performance for the different ESC tasks. Consequently, the question arises whether the different measures of ESC performance are interrelated. For this reason, we computed pairwise correlations between the dichotomous variables for the bar-transport-task and the sword-rotation-task and the continuous variable for grasp height differences (see Table 3)^4^.

**Table 3:** **Pairwise correlations between the three motor tasks**.

	**Correlations between the motor tasks**		
**Age**	**Bar-transport-task and sword-rotation-task**	**Bar-transport-task and grasp-height-task**	**Sword-rotation-task and grasp-height-task**
3-year-olds	−0.258	−0.144	−0.155
4-year-olds	0.565^**^	0.263	−0.113
5-year-olds	0.283	0.210	0.126
6-year-olds	0.214	0.247	0.261
7-year-olds	0.055	0.079	0.236
8-year-olds	0.071	0.071	0.020
9-year-olds	0.233	−0.115	−0.326
10-year-olds	−0.125	−0.058	0.386
Adults	N/A	0.367	N/A
Overall	0.276^**^	0.305^**^	0.245^**^

*that two correlations for the adult sample could not be computed due to ceiling effects in the sword-rotation-task*.

** *p < 0.001*.

Small to medium correlations emerged when considering the entire sample (last row of Table 3), indicating an increase in ESC sensitivity as age increases, even though these correlations are mostly driven by the between-group differences described above. By contrast, there was no sign of intercorrelations of the different ESC measures within the groups (except for the correlation of the bar-transport-task and the sword-rotation-task for the 4-year-olds). To further assess these within-group differences, we Z-transformed each correlation coefficient for each of the non-adult groups, averaged these transformed values and re-transformed the resulting values to correlation coefficients. This procedure yielded mean correlations of *r* = 0.14 for the bar-transport-task and the sword-rotation-task, *r* = 0.07 for the bar-transport-task and the grasp-height-task, and *r* = 0.06 for the sword-rotation-task and the grasp-height-task. Testing the corresponding mean *Z*-values against zero did not yield any significant differences, *p*s > 0.193.

### The relationship between ESC and EF

As in the three ESC tasks, children also improved their performance in the three EF tasks with increasing age. However, as indicated in the Data Analysis, it was not possible to analyze this trajectory due to the usage of different task versions (e.g., a 7-year old child performed numerally “poorer” on the TOH task, but only because of a switch in task versions from 6 to 7 years, with 7-year-old performing a more difficult version than younger children)^5^. The main purpose of this study was to assess a possible relationship between motor planning and executive functioning. Therefore, Pearson correlations were computed between all motor (dichotomous variables for the bar-transport-task and the sword-rotation-task and continuous variables for the grasp-height-task) and cognitive tasks (see Table 4).

**Table 4:** **Pairwise correlations between the motor and the cognitive tasks**.

	**Correlations between the cognitive and motor tasks**								
	**D2-task**			**Mosaic-task**			**Tower of Hanoi**		
**Age**	**BTT**	**SRT**	**GHT**	**BTT**	**SRT**	**GHT**	**BTT**	**SRT**	**GHT**
3-year-old	−0.02	0.078	0.334	0.239	0.075	0.257	−0.039	0.033	−0.126
4-year-old	−0.032	0.207	0.071	0.068	−0.344	0.272	0.414^*^	0.004	0.356
5-year-old	0.358	0.19	0.492^*^	0.279	0.278	0.406^*^	0.112	−0.201	−0.06
6-year-old	0.064	0.129	−0.046	0.059	0.165	−0.112	0.072	0.072	0.053
7 -year-old	0.049	−0.104	−0.263	0.261	−0.084	−0.011	0.053	−0.325	−0.184
8 -year-old	0.137	−0.043	−0.078	0.16	0.418^*^	−0.101	−0.156	0.179	−0.159
9-year-old	0.173	−0.133	0.427^*^	0.04	0.04	−0.206	−0.018	0.231	0.064
10-year-old	0.018	−0.048	0.384	0.57^*^	0.11	0.046	0.059	0.103	−0.061
Adults	0.003	N/A	−0.181	0.11	N/A	0.124	0.038	N/A	0.065

*BTT, Bar-transport-task; SRT, Sword-rotation-task; GHT, Grasp-height-task*.

* *p < 0.05*.

The analyses yielded mostly small and non-significant correlations across the participants of each individual group (for exceptions see Table 4).

In contrast to the ESC tasks, overall correlations across the participants of different groups are not possible for the EF tasks, because we opted to use different versions of the tasks for different age groups. In other words: As cognitive function develops during childhood, it was not possible to use only one test for every single cognitive function. In all tasks, two or three different versions of each test were used to examine executive functions as described in the methods section. As for the intercorrelations of the ESC tasks, however, we computed mean correlations across all non-adult groups and tested the resulting mean Z-score against zero. This procedure yielded a significant correlation only between the mosaic task and the bar-transport-task, *r* = 0.218, *p* = 0.018, whereas the remaining correlations were not significant *p*s > 0.082.

## Discussion

The goal of the present study was three-fold: (1) to examine the developmental trajectories of the different (ESC) tasks, (2) to assess possible relationships between all motor tasks used, and (3) to investigate potential relations between the performance in both, the motor and the cognitive task, based on the hypothesis of an association between EFs and ESC planning. To this end, a specific test battery examined the development of motor planning abilities and executive functions in children and adults. To examine motor planning abilities, the bar-transport-task, the sword-rotation-task, and the grasp-height-task were conducted. To test for EF, we used the Tower-of-Hanoi-task, the Mosaic-task, and the D2-attention-endurance-test. This test battery was employed to assess the performance of eight groups of children, aged 3–10 years, and one group of adults.

With regard to the developmental trajectories observed for motor planning abilities (as indicated by the ESC effect), the results support previous studies using the bar-transport-task (Hughes, 1996; Smyth and Mason, 1997; Thibaut and Toussaint, 2010; Weigelt and Schack, 2010; Jovanovic and Schwarzer, 2011; Knudsen et al., 2012; Stöckel et al., 2012) and the sword-rotation-task (Crajé et al., 2010; Jongbloed-Pereboom et al., 2013). Specifically, the likelihood to perform a certain motor action in an ESC-consistent manner (steadily) increased from young kindergarten children to school children and from school children to adults. For the bar-transport task, adult-like performance was reached around the age of 10 years (Thibaut and Toussaint, 2010; Stöckel et al., 2012; see also Knudsen et al., 2012; Stöckel and Hughes, 2016). For the sword-rotation task, this development seems to be somewhat delayed, as even 10-year old children showed the ESC effect less often than adults (see also Jongbloed-Pereboom et al., 2013). In addition, the results of the grasp-height-task suggest that this basic developmental trend across young ages generalizes from a rather dichotomous grip selection (underhand vs. overhand) to grip choices in a continuous task space (here, along the vertical axis of the object, for a different continuous ESC paradigm, see Herbort and Butz, 2012). However, participants appear to display adult-like performance much later in these kinds of tasks, as only about half of the 10-year old children showed the ESC effect in the grasp-height task. Here it should be noted, however, that only 50% of the adults grasped the plunger in accordance with the ESC effect.

The developmental pattern of motor planning abilities across the different age group, as signified by the ESC effect, did not follow a strictly linear trend. In both, the bar-transport-task and the sword-rotation-task, a stagnation in the developmental trajectory of ESC planning could be observed. These findings are in line with previous studies, and they are commonly explained in terms of the motor re-organization hypothesis (Bard et al., 1990; Thibaut and Toussaint, 2010). According to this hypothesis, motor structures re-organize in children around the age of 8, resulting in a momentary instability of previously acquired abilities. It is likely that different sensory-motor maturation processes, which support the development of cognitive control during early childhood (Piaget and Cook, 1952), also enable the development of ESC planning (Fischer, 1980). Interestingly, 8-year-old children provided less evidence for anticipatory planning than 6-year-old children in the study of Thibaut and Toussaint (2010), 7-year-old less than 6-year-old children in the study of Jongbloed-Pereboom et al. (2013). The developmental trajectories of the present study can therefore be taken to support the motor re-organization hypothesis, as children aged 7 and 8 years showed a stagnation (or even a decrease in the grasp-height-task) in the development of their motor planning abilities.

As for the second goal of the study, the correlation analyses did not provide much support for intercorrelations between the three motor tasks. Although, correlations emerged when analyzing the data across the entire sample, no such relationship was found within any of the different age groups (with only one exception). The absence of any correlation between the motor tasks is surprising, but is also in line with three other previous studies^6^ testing children in two motor planning tasks within a single experiment (Smyth and Mason, 1997; Knudsen et al., 2012; Stöckel and Hughes, 2016). Please note that in all other studies conducted so far, only a single motor task was used. The current work therefore extends this previous line of research and is the first to investigate the development of motor planning abilities by using three different tasks in a within-subjects design. There are several possible reasons why children's performance in the three motor tasks was not correlated. The tasks to measure ESC planning abilities might have been too different to exhibit relationships between their developmental patterns. They differed regarding many details, like the number of required action steps, the precision requirements, children's perception of comfort, the required degree of object rotation, children's familiarity with the task, and/or motivational aspects (for a more detailed discussion of these influencing factors see Wunsch et al., 2013). All of these aspects (or combinations of it) may have prevented intercorrelations between these three tasks to occur. However, it is also possible that the performance in each of these ESC tasks is not based on a common set of motor planning abilities. This notion should certainly be considered in future research.

As for the third goal of the study, we also did not observe any conclusive evidence for interdependencies between the development of ESC performance and EF. Mean correlations within groups of children of similar age were either absent or small. The notion that motor skill development may not be as closely related to the maturation of EF has been previously assumed and put forward by Van Swieten et al. (2010). These authors argued that if adults do not always perform in a manner consistent with ESC, then EF must fail in these cases. However, it is also plausible that EF may sometimes fail under certain circumstances, even in adults, as has been shown, for example, by Blakemore and Choudhury (2006), De Luca et al. (2003) or Salthouse et al. (2003). Alternatively, it might be that the selection of EF tasks may not have been appropriate to examine a relationship to ESC planning. Maybe, the EF tasks chosen for this study (i.e., problem solving, visual perception, and attention) are not appropriate to measure intercorrelations with motor planning abilities. Noteworthy, Stöckel and Hughes (2016) found response planning/problem solving in the Tower of London task to be a significant predictor of anticipatory motor planning performance for a group of 5- to 6-year old children. This suggests that other tests to assess EF than have been used inthe present study should be considered in the future. Moreover, the relatively small number of participants in each age-group is a limitation of the present study. Findings within each group should thus be treated with caution, whereas there is good reason to interpret the overall finding of a null-correlation as evidence for a true null effect (rather than a Type II error).

The observation of largely independent processes underlying EC and ESC also seems to challenge the notion of embodied accounts of cognition and action. At the same time, these findings seem to resemble the concept of simulators (rather than simulations), which hold a prominent spot in embodied theorizing (Barsalou et al., 2003). Such simulators are mechanisms that are assumed to provide context-specific simulations and they have been invoked as an embodied alternative to the term of a “concept” (Barsalou, 2003). Our findings therefore suggest that different motor requirements as used in the current bar-transport-task, sword-rotation-task, and grasp-height-task actually rely on rather different simulators to represent the corresponding actions.

## Conclusion

In summary, the present study examined children's performance in three object manipulation tasks and compared their performance with three cognitive tasks that measured EF. There was a clear developmental trajectory for all abilities examined here, and this trend occurred in a similar fashion for all motor planning tasks. Contrary to our predictions, however, the findings showed only weak and unreliable intercorrelations between the different motor tasks. In addition, the performance in the cognitive tasks used to test EF did not reliably predict participant's performance in the different ESC tasks. Future research is needed to further assess potential interdependencies between motor skill development and the maturation of cognitive abilities. Specifically, the current findings suggest that motor planning is a rather heterogeneous ability that cannot be captured by one single task.

## Author contributions

All authors made substantial contributions to the conception, analysis and interpretation of results; they drafted and revised the manuscript critically for important intellectual content and approved the final version of this manuscript to be submitted. They all agree to be accountable for all aspects of the work in ensuring that questions related to the accuracy or integrity of any part of the work are appropriately investigated and resolved.

## Funding

The article processing charge was funded by the German Research Foundation (DFG) and the Albert Ludwigs University Freiburg in the funding program Open Access Publishing.

### Conflict of interest statement

The authors declare that the research was conducted in the absence of any commercial or financial relationships that could be construed as a potential conflict of interest.

## Footnotes

^1^For each age group, problem difficulties were as follows, whereby the first number always indicates the number of discs used, the second number always indicates the number of moves for optimally solving the problem: 3-2, 3-3, 3-4, 3-5, 3-6, 4-7, 4-8, 4-9, 4-10, 4-11, and 4-15 for 3- and 4-year olds; 3-4, 3-5, 3-6, 4-7, 4-8, 4-9, 4-10, 4-11, 4-13, 4-15 for 5- and 6-year olds; and 4-7, 4-9, 4-10, 4-11, 4-13, 4-15, 5-20, 5-24, 5-27, and 5-31 for children older than 7 years and adults.

^2^The Mosaic-task is a subtest of the HAWIVA^®;^-III [Hannover Wechsler Intelligenztest für das Vorschulalter; German translation and adaption of the WPPSI^®;^-III (Wechsler Primary and Preschool Scale of Intelligence) of David Wechsler (2002); Ricken et al. (2007); for preschool children], the HAWIK^®;^-IV [Hamburg Wechsler Intelligenztest für Kinder; German translation and adaption of the WISC^®;^-IV (Wechsler Intelligence Scale for Children) of David Wechsler (2003); Petermann and Petermann (2008); for school children] and the WIE^®;^ [Hamburg Wechsler Intelligenztest für Erwachsene; German translation and adaption of the WAIS^®;^-III (Wechsler Adult Intelligence Scale) of David Wechsler (1997); Von Aster et al. (2006); for adults] to assess visuospatial and motor skills.

^3^We measured different dependent variables for the Mosaic and the Tower of Hanoi tasks, e.g. the total amount of correctly solved items, the total score and the score with bonus-points for fastest resolve in the Mosaic task, and the amount of incorrect moves, the number of correctly solved items or the number of the item where the first mistake was made before doing the first move in the Tower of Hanoi task. Due to their correlations (Mosaic-task: *r* = 0.229 for correctly solved items and total score; *r* = 0.194 for correctly solved items and bonus-score, and *r* = 0.950 for total score and bonus-score; all *p* < 0.01; Tower-of-Hanoi-task: *r* = 0.261 for the amount of incorrect moves and the item with the first mistake, *r* = 0.149 for the amount of incorrect moves and the number of correctly solved items, and *r* = 0.218 for the item with the first mistake and the number of correctly solved items; all *p* < 0.05), however, we chose the most superordinate variable for further analysis.

^4^Following a suggestion of a reviewer, we verified all bivariate correlations for both ESC tasks with binary outcome measures, i.e., the bar-transport task and the sword rotation task, with the analogous test of the phi coefficient against the X^2^-distriubtion (with $t_{Pearson}^{2}=\frac{\left(n-2\right){\text{X}}^{2}}{n-{\text{X}}^{2}}$). Also, for both tasks, we evaluated the relation to measures of EF via the glmer function of the lme4 package in R. Results mirrored the correlation analyses reported in Tables 3, 4.

^5^For further information on EF results please contact the corresponding author.

^6^As the first author of this paper was a co-author in the study by Knudsen et al. (2012), data was checked *post-hoc* for relations between the two ESC tasks used in either study (the grasp-height-task was excluded due to the continuous nature of the dependent variable). Phi-coefficient analyses revealed similar results: In the study by Knudsen et al. (2012), the bar-transport-tasks and the overturned-glass-task was only correlated in 4-year-old children (ϕ = 0.522, *p* < 0.05), In our study, however, we found both tests to be correlated in the 4- and 5-year-olds (ϕ = 0.476, *p* < 0.01 and ϕ = 0.440, *p* < 0.01), but not in the other participant groups. This means, that the two tasks used in our study and the two tasks used in the study by Knudsen et al. (2012) possibly measure different aspects of motor planning. Hence, the developmental trajectories of ESC planning may not be related between these different tasks, possibly due to different task constraints, as already discussed in Wunsch et al. (2013).
